# Impact of Anthropogenic Activities and Climate Change on the Wetlands in the North‐West of Madagascar

**DOI:** 10.1002/pei3.70206

**Published:** 2026-08-29

**Authors:** Botovao Auguste Ramiandrisoa, Jenny Thérèsianne Raoelina, Solombolatiana Tsiarosarobidy Ramanandraibe, Nadiah Vololomboahangy Manjato, Cyrille Maharombaka, David Goyder, Hery Lisy Tiana Ranarijaona

**Affiliations:** ^1^ Doctoral School Natural Ecosystèms University of Mahajanga Mahajanga Madagascar; ^2^ Faculty of Sciences, Technologies and Environment (FSTE) University of Mahajanga Mahajanga Madagascar; ^3^ Missouri Botanical Garden (MBG) Antananarivo Madagascar; ^4^ Royal Botanic Gardens Kew Richmond UK

**Keywords:** Boeny, conservation, disturbance, Macrophytes, wetlands

## Abstract

Macrophytes are vulnerable to various factors in their natural ecosystems, including climatic, ecological, and anthropogenic changes. This study aims to document how these factors influence floristic composition. Four sites were investigated in the Boeny Region of northwest Madagascar: Ambatoboeny, Marovoay, Ankarafantsika National Park, and Mahajanga II. Floristic inventories were compiled with geographic and floristic parameters, utilizing 1 × 1 m^2^ quadrat for each sample. Diversity indices, Analysis of variance (ANOVA), distance based‐Redundancy Analysis (db‐RDA) were conducted to analyze the data. A total of 98 species were recorded across the four study sites, belonging to 73 genera and 33 families. Twelve species were unique to Mahajanga II, 18 to Marovoy, 16 to Ankarafantsika, and five to Ambatoboeny. Rarefaction curves indicated a progressive trend in species diversity, which appeared to stabilize in both Shannon diversity. The db‐RDA revealed species dispersion along longitudinal and latitudinal axes and in relation to vegetation cover. The Permutational Multivariate Analysis of variance (PERMANOVA) indicated a significant influence among the study sites. Over the course of two decades, we observed an increase in temperature at the three districts including the study sites, while precipitation levels progressively decreased during the same period. Wetlands in the northwestern part of Madagascar are in need of conservation and would benefit from further research.

## 1 Introduction

Both terrestrial and marine wetlands are vulnerable ecosystems worldwide. During the 20th century, these ecosystems experienced a decline in both extent and quality of approximately 75% (Gardner et al. 2015). Lakes, rivers, ponds, and other types of wetlands are particularly exploited by humans. However, wetlands store about 12% of the global carbon pool, playing a significant role in the global carbon cycle (Erwin 2009).

Anthropogenic activities, especially the conversion of wetlands to paddy fields, pose major challenges to these ecosystems (Gardner et al. 2015; Maharombaka et al. 2017; Phillipson et al. 2018). In fact, biological diversity suffers due to various factors, including pollution, the invasion of alien species, and overexploitation (Gardner et al. 2015; Pittock et al. 2015). The increase in settlements contributes highly the rate of pollution, particularly affecting the rivers (e.g., De Andrade Costa et al. 2020). Mining is one of the most serious threats to water quality and aquatic life, as it can release heavy metals and reduce biological diversity (Phillipson et al. 2018; Rico‐Sánchez et al. 2022). Invasive freshwater species create significant challenges for biodiversity, especially for endemic and threatened species (Odelu et al. 2014). The wetlands of Madagascar have been degraded in over 60% since 1960 (Madagascar—Restoring Lake Sophia | WWT n.d.).

The impact of climate hazards contributes to the decline of wetlands and the loss of biodiversity. This phenomenon affects the behavior of invasive species and leads to their increased prevalence over time (Hrivnák et al. 2019). Madagascar is one of several countries with terrestrial wetlands of international importance. The island currently has 21 Ramsar sites, covering more than 2.1 million hectares, recognized for their exceptional biodiversity and ecological importance. The study sites are included in the KBAs zone in the north‐west of Madagascar (MDG‐224: Mahajanga Costal Zone; MDG‐141: Ankarafantsika National Park; MDG‐211: Maevatanana‐Ambatoboeny wetlands) (CEPF 2022). In addition to the wetlands located within protected areas, the island also has numerous small bodies of wetlands, some of which are permanent while others are temporary. Most of them are interconnected during the rainy season and dry out during the dry season (temporary wetlands). These areas are home to a variety of aquatic species, including both fauna and flora. However, wetlands can be disturbed by various factors such as natural and anthropogenic activities.

The north‐western part of Madagascar falls within a semi‐arid bioclimatic zone and is characterized by dry semi‐deciduous forests (Rajeriarison and Faramalala 1999; Moat and Smith 2007). The ecology of aquatic plants in Madagascar, particularly in the northwest, has been largely understudied. Climatic hazards can impact wetlands areas and subsequently affect the floristic composition due to anthropogenic activities.

The objectives are to document the floristic composition of macrophytes (The macrophytes are aquatic photosynthetic organisms, large enough to see with the naked eye, that actively grow permanently or periodically submerged below, floating on, or up through the water surface). (i) under the influence of various human activities; (ii) in the context of climate change. Specifically, we aim to (1) inventory the macrophytes and characterize the areas they occupy, and (2) study the ecology of the macrophytes within the selected sites. The hypotheses have been proposed to characterize the influence of the species and the trends occurring in the wetlands: (i) the long dry season is associated with an increase in temperature and a decrease in rainfall; (ii) human activities impact the presence and distribution of aquatic flora, as well as the overall ecology of the wetlands.

## 2 Materials and Methods

### 2.1 Study Site

Characteristics of the sites: The Boeny Region spans a total area of 29,826 km^2^ and is divided into six administrative zones, all located at an altitude below 800 m (CREAM 2013). Our study sites are situated within the districts of Ambatoboeny (8028 km^2^), Mahajanga II (4721 km^2^), and Marovoay (5629 km^2^).

The Ambatoboeny site is located at 46°40′27.7″ E; 16°28′13.6″ S, while Marovoay is at 46°39′46.1″ E; 16°8′5.8″ S. Both sites are in the lowlands and are connected by the Betsiboka River. The soil in these areas is primarily red lateritic and derived from the catchment basin. Ankarafantsika National Park, located at 46°49′13″ E; 16°16′10.6″ S. The soil is characterized as sandy sandstone. The district of Mahajanga II is located at 46°54′31.5″ E; 15°38′36.7″ S, and also featured red lateritic soil.

In Mahajanga II, Marovoay, and Ambatoboeny, land use is predominantly agricultural (CREAM 2013). Overall, the average annual temperature in the region is 31°C, with an average annual rainfall of 1338.6 mm.

**FIGURE 1:**
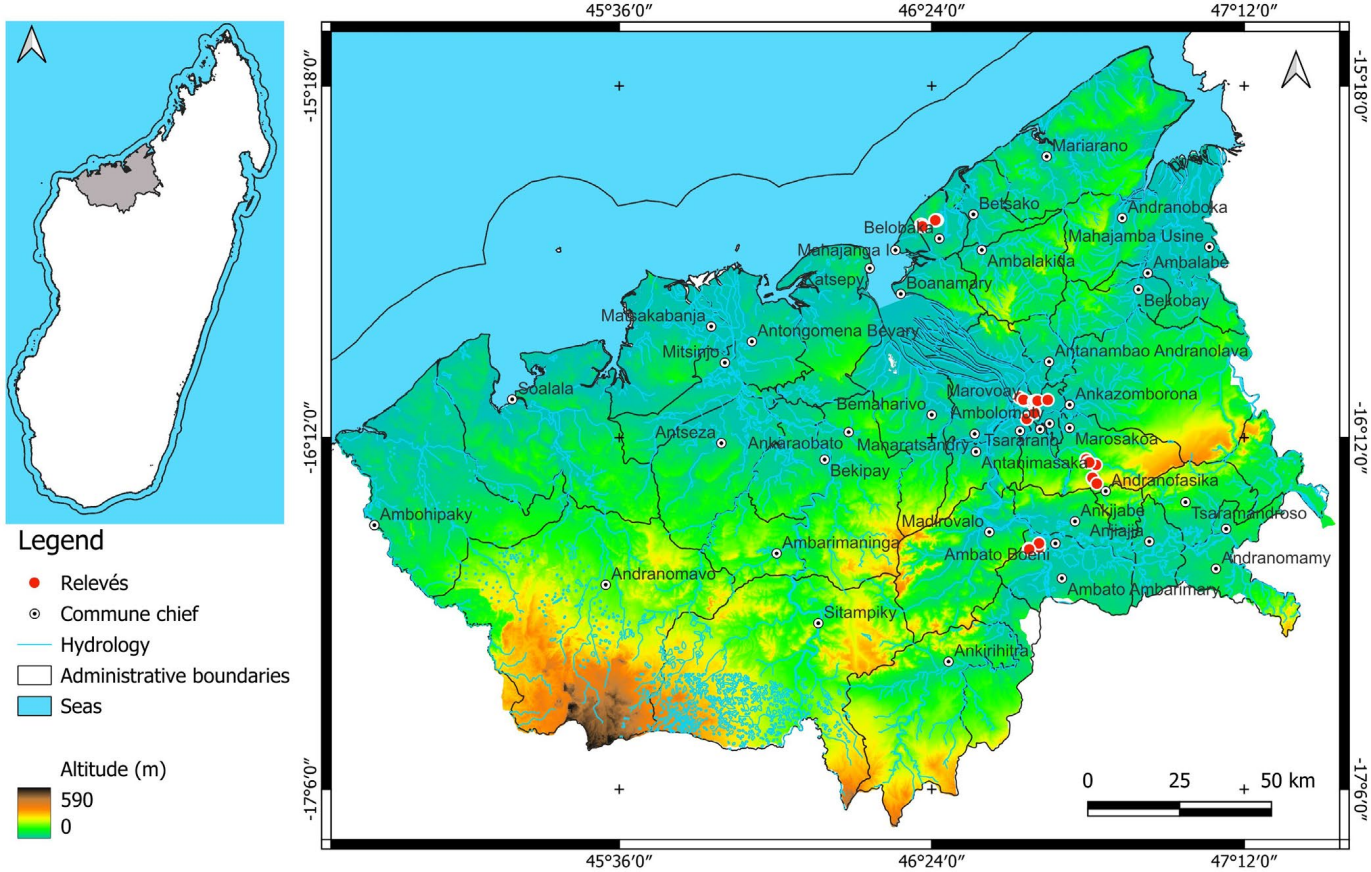
Map representing the study site from the three districts such as Marovoay, Ambatoboeny, and Mahajanga II.

Choice of site: This study focuses on four areas (Figure 1), one protected area (Ankarafantsika National Park) and three non‐protected sites (Ambatoboeny, Marovoay, and Mahajanga II) where wetlands have not been designated as protected. The first site, which includes herbaceous marshes and a lake, is located within the protected area. In contrast, the other three sites are outside the protected area, where agricultural conversion and human activities are prominent. In addition, Ambatoboeny and Marovoay are agricultural vocations. For Mahajanga II, the catchment of the wetlands is converted for small agriculture. This reason led us to select these sites for our study to compare their state. However, despite the protections afforded to Ankarafantsika National Park, human activities in the surrounding locations are contributing to the decline of the wetlands and the wildlife that depend on them.

### 2.2 Data Collection

Data were collected over a period of 2 months (March–April 2022) during the dry season across four different sites. A total of 74 quadrats (1 × 1 m^2^) were established in various zones, including catchment areas, canals, ponds, lakes, marshes, and rice fields. The type of zones present in each sites are as follow: Catchment, Ponds and rice field were investigated at Ambatoboeny, Lake, Marshes, and Rice field at Ankarafantsika nation park, Marshes, Ponds, and Rice field at Mahajanga II, Canal, and Rice field at Marovoay. Floristic and ecological parameters were recorded based on abundance dominance indices (+, r, 1, 2, 3, 4 and 5), following the Braun‐Blanquet method (Guinochet 1973). Additionally, total vegetation cover and height were measured. Voucher specimens were collected for subsequent identification and are deposited at the national herbarium at TAN (Parc Botanic et Zoologique de Tsimbazaza, Antananarivo) and at the Ecole Doctorale Ecosystèmes Naturels (EDEN), University of Mahajanga. These voucher specimens were placed in the old journal paper and dried in the ambient air.

### 2.3 Biological Types

The biological types were characterized using the Raunkiaer method (Sirvent 2020).

### 2.4 Data Analysis

The overall analysis was conducted using R version 4.2.2 (Core Team 2023). To obtain the number of common and shared species in each location, a Venn diagram was used from the ‘*ggvenn*’ function (‘*ggvenn*’ package). This analysis allow us to visualize the similarity or differences between site. We used the ‘*upset*’ function from ‘*ComplexUpset*’ to characterize the biological types from each area. This function can handle a large set of groups and also characterize other data qualification from the inner and the interaction site.

A rarefaction curve was generated to analyze the trends and variations in biodiversity at the study sites. We also performed interpolation and extrapolation of the Hill numbers for the recorded species. Additionally, we estimated specific richness (*q* = 0), Shannon diversity (*q* = 1). The *iNEXT* function from the *iNEXT* package (Hsieh et al. 2016, 2022) was used for these analyzes.

A correlation analysis was conducted on the environmental variables to verify the presence of multicollinearity in the data matrix. Following this, a multivariate analysis was performed to assess the influence of the distribution of the relevés and species. The raw abundance dominance data was transformed using the ‘*Hellinger*’ method. Two data matrices were analyzed simultaneously (one for relevés × species and another for relevés × environmental factors).

This analysis was carried out using distance‐based redundancy (dbRDA) and applying the Bray‐Curtis distance (Legendre and Anderson 1999; McArdle and Anderson 2001; Legendre and Legendre 2012). To further explore any differences, a multivariate permutation analysis (PERMANOVA) was conducted using the *adonis2* function from the «*vegan*» package (Anderson 2001), which is a non‐parametric analysis. If the site factors demonstrated significant statistical differences, a pairwise comparison analysis was performed using *the pairwise.adonis* function from the ‘*pairwise.Adonis2*’ package with 9999 times permutations. Lastly, the trend of the time series in the temperature and rainfall data was analyzed with the ‘*ts’* function (Kendall and Stuart 1983) from the ‘*stats*’ package, while the trend was further assessed using the ‘*decompose*’ function from the same package.

## 3 Results

### 3.1 Species Richness

A total of 98 species from 73 genera and 33 families were recorded across the four study sites (Supporting Information S1). The most represented families were Cyperaceae (21 species; 21.4%), Poaceae (20 species; 20.4%) and Fabaceae (12 species; 12.2%). The Malvaceae was represented by four species (4.1%) while Commelinaceae and Onagraceae each had three species (3.1%).

**FIGURE 2:**
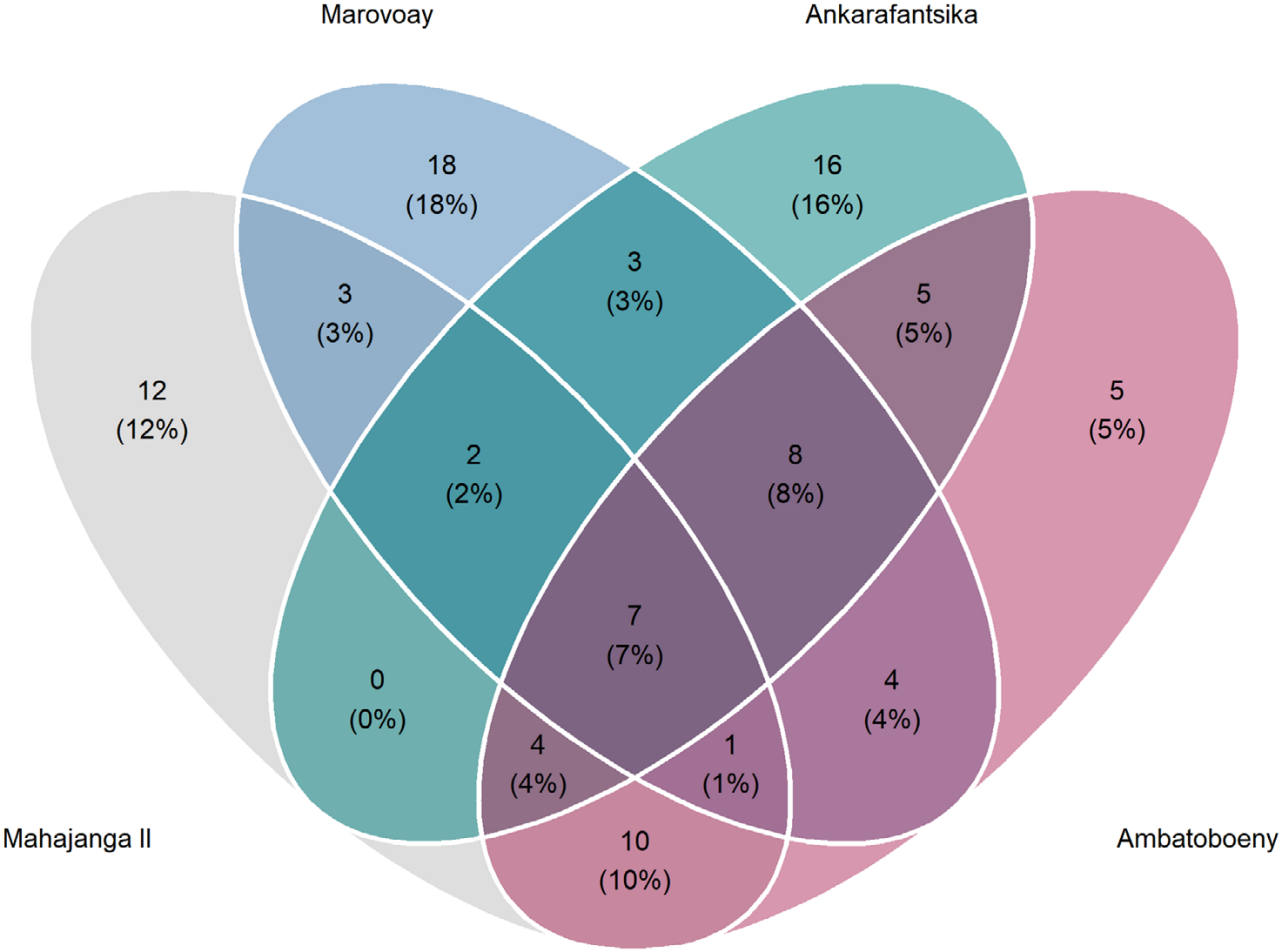
Venn diagram representing the number of species which occur in each site and the common species in the four sites such as Mahajanga II, Marovoay, Ankarafantsika, and Ambatoboeny.

According to Figure 2, Marovoay has 46 species, 18 of which are unique to that location, followed by Ankarafantsika with 45 species, 16 of which are unique. Ambatoboeny recorded 44 species, five of which are its own, and Mahajanga II had the fewest with 39 species, 12 of which are unique. The number of genera reflected a similar pattern to that of the species. According to the Supporting Information S2, the mean number of species varied between 3.5 species, found in the marshes and rice fields at Mahajanga II, and eight species, found in the rice fields at Ambatoboeny.

### 3.2 Diversity Indices

**FIGURE 3:**
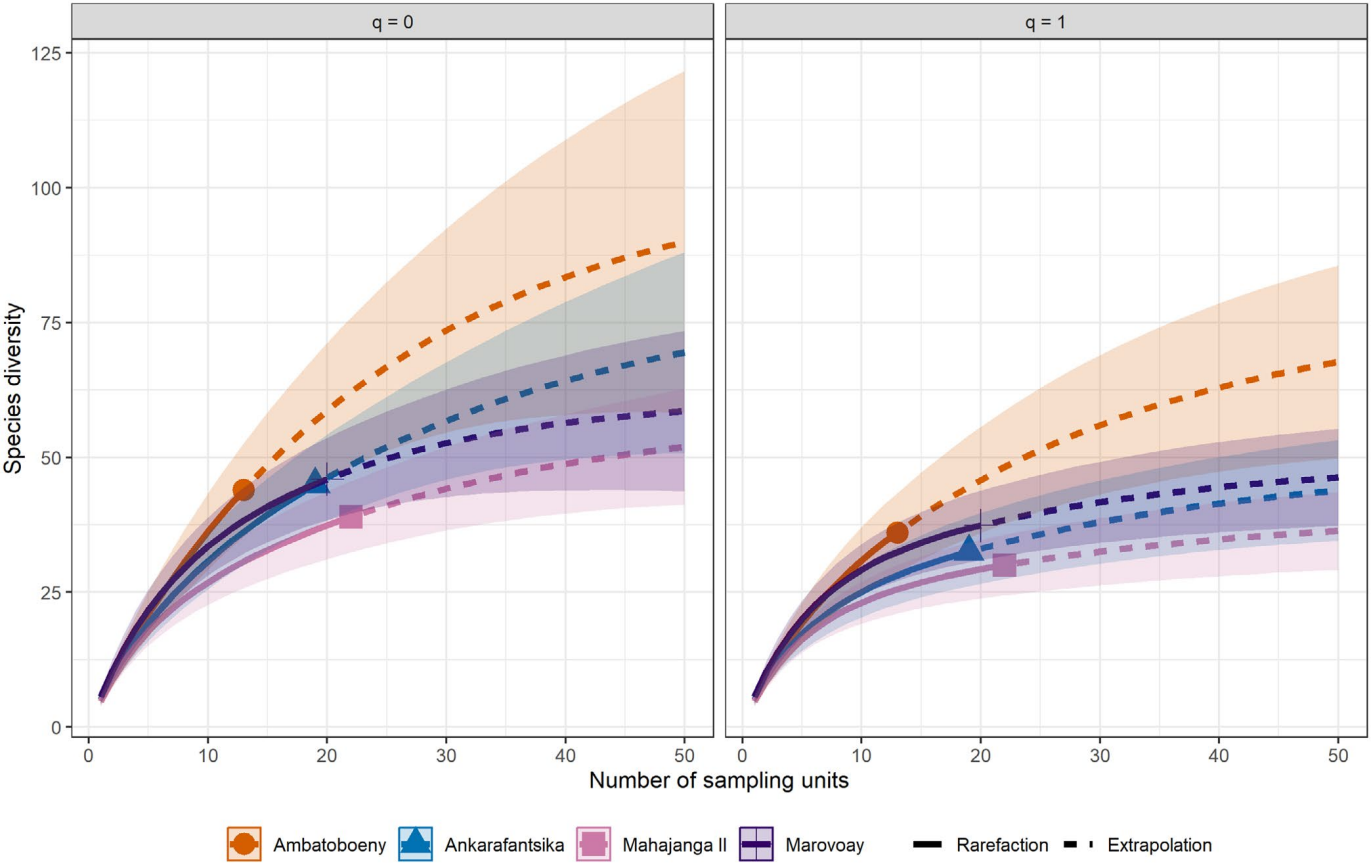
Observed and estimated species diversity following the Hill diversity (*q* = 0: Specific richness; *q* = 1: Shannon diversity). The curve ribbon indicates the Confidence Interval (CI) at 95%. The solid trait shows the observed species and diversity of Shannon. The dots represent the extrapolation of the estimated values. The curves tend to increase indicating the lack of sample size particularly in the Ambatoboeny site. The number of species is higher at Ambatoboeny and lower at Mahajanga II, and the same for the Shannon diversity.

The rarefaction curves (Figure 3) indicate a trend of increasing specific diversity (*q* = 0). Among the study sites, Ambatoboeny had the highest species estimation (Chao: 103 ± 30; Confidence Interval (CI) at 95%: 67–197), followed by Ankarafantsika national park (Chao: 82 ± 20; CI at 95%: 58–147), then at Marovoay (Chao: 61 ± 9; CI at 95%: 51–93), and the lower species estimation was recorded at Mahajanga II (Chao: 58 ± 13; 95% CI: 45–102).

The Shannon diversity (*q* = 1) also showed a positive trend at Ambatoboeny, where it reached 36.078 bits (estimated at 78.006 ± 12.444). However, Marovoay had an observed value of 37.419 (estimated at 49.695 ± 3.886); then Ankarafantsika national park had 32.547 (estimated at 49.652 ± 6.808), and the lower Shannon diversity was found at Mahajanga II with 30.051 (estimated at 40.084 ± 4.115).

### 3.3 Species Distributions

In the overall study, native species present the majority, with 97 species recorded (98.97%). In contrast, the endemic species are represented by only three species (3.06%) such as *Melochia betsiliensis* Baker, *Panicum luridum* Hack. ex Scott‐Elliot, and *Crinum firmifolium* Baker. However, Ambatoboeny has two species *C. firmifolium*, and *M. betsiliensis*. Mahajanga II, also, has two species such as *P. luridum* and *C. firmifolium*. Ankarafantsika and Marovoay had only one each (*C. firmifolium* and *M. betsiliensis*, respectively).

### 3.4 Biological Types

**FIGURE 4:**
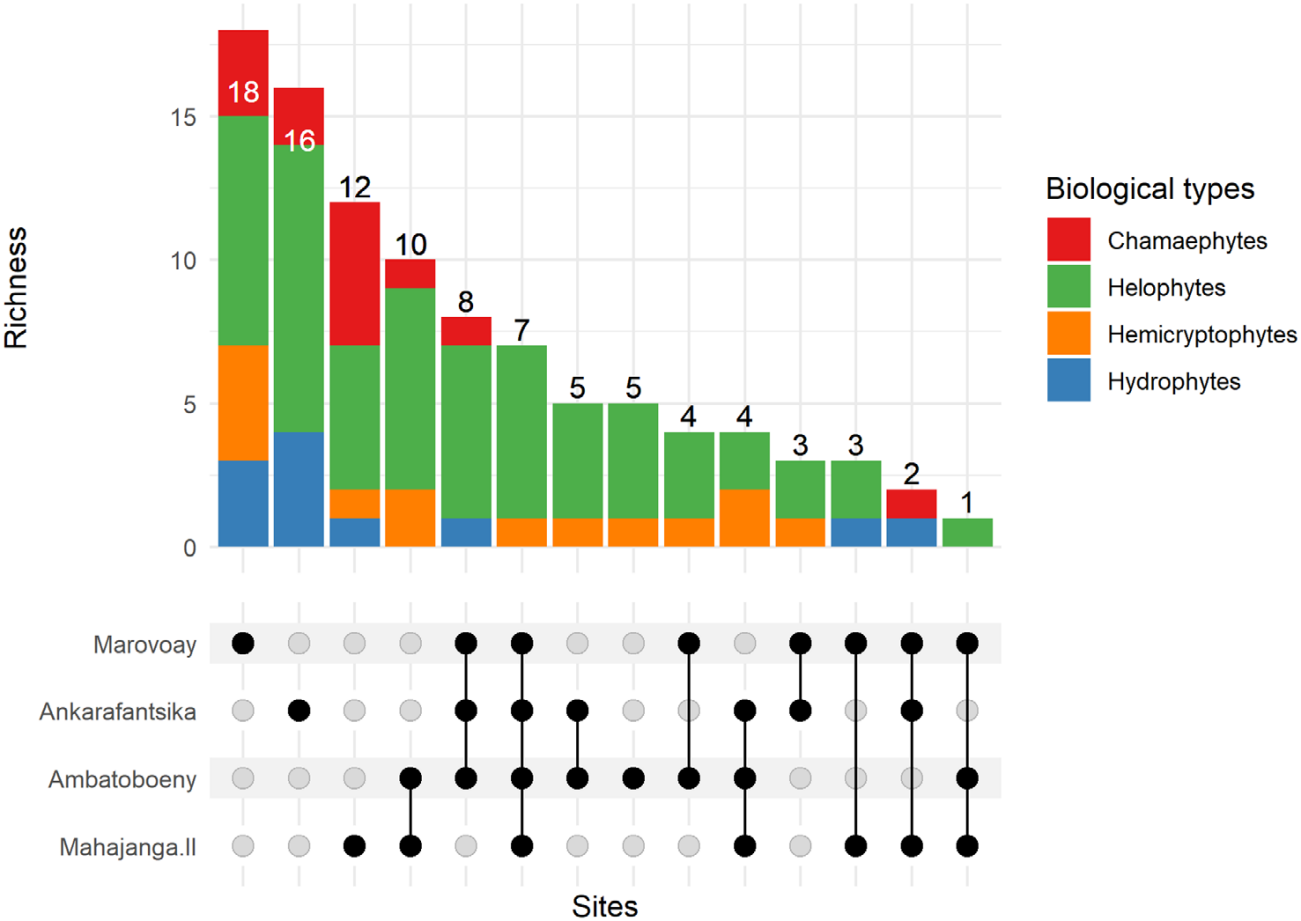
Representation of biological types that characterize the study area. The single dot shows the biological type of the unique site. The interaction dots represent the biological type in common species. The number in each bar represents the number of species in each site and their interactions.

Following the Raunkiaer classification (Figure 4, Supporting Information S3), helophytes had the highest proportion (61.2%, 60 species), followed by hydrophytes (11.2%, 11 species). However, other types were recorded in the list. There are especially the species belonging to the hemi‐cryptophytic group localized in the banks of the wetlands (14.3%; 14 species), and chamaephytes (13.3%; 13 species).

### 3.5 Influence of Floristic Distribution

The non‐parametric Spearman correlation analysis was conducted to examine the relationship between the floristic variables (Pielou indices and vegetation Cover) and geographical variables (Lat: Latitude; Long: Longitude), as summarized in Table 1.

**TABLE 1:** Spearman test (non‐parametric correlation) between the floristic and geographic variables (alpha = 0.05).

	Variables	Cover	Long	Lat	Pielou
*rho*	Cover		0.53^ns^	0.11^ns^	0.68^ns^
	Long	−0.07		**< 0.001** ***	**0.014** *
	Lat	−0.18	**−0.64**		0.655^ns^
	Pielou	0.05	**−0.28**	0.05	

*Note:* ns show the non significant test between variables. The values colored in light‐gray are the coefficient of correlation and in the dark‐gray are the probabilities.

Abbreviations: Cover, Vegetation cover; Lat, Latitude; Long, Longitude; Pielou, Pielou Index.

* Low significant test.

*** Highly significant test.

The result indicated that the vegetation cover was not significantly correlated with longitude (Long; *ρ* = −0.07; *p* = 0.53), latitude (*ρ* = −0.18; *p* = 0.11), nor with the Pielou indices (*ρ* = 0.05; *p* = 0.68). In contrast, a significant negative correlation was found between latitude and longitude (*ρ* = −0.64; *p* < 0.001), as well as between the Pielou indices and longitude (*ρ* = −0.28; *p* = 0.014). It indicated that the location of the sites in Ankarafantsika, Ambatoboeny, and Marovoay is located more inside the island (i.e., far from the coastal side) than the Mahajanga II. These findings helped identify any potential collinearity among the variables, and it was determined that none exhibited this phenomenon. Therefore, we proceeded to use all the variables for distance‐based redundancy analysis using the Bray‐Curtis method.

The analysis was conducted using distance‐based redundancy (dbRDA) according to the Bray‐Curtis method. This analysis revealed a dispersion of the relevés and species across different locations. The geographical variables demonstrated highly significant differences according to the analysis of variance (ANOVA, 9999 times of permutations; *F* = 3.94, *p* = 0.0001 for longitude, and *F* = 1.78; *p* = 0.001 for latitude). Additionally, the floristic variables also exhibited a significant difference (ANOVA, vegetation cover, *F* = 1.96; *p* = 0.0002). The axes revealed a highly significant correlation with both dbRDA1 and dbRDA2 (*F* = 4.25; *p* = 0.0001, and *F* = 2.09, *p* = 0.0002, respectively).

The first two axes explained 70.4% of the total variances. The first axis of the distance‐based redundancy analysis (dbRDA1: 47.2%) was characterized by the distribution of the relevés occurring at Ankarafantsika site in the negative side, while the Mahajanga II relevés was positioned in the positive side, corresponding to geographical gradients (longitude and latitude, respectively). The species that influenced the axis on the negative side included *Pycreus mundtii* Nees, *Fuirena umbellata* Rottb., *Cyperus prolifer* Lam., and *Ludwigia octovalvis* (Jacq.) P.H. Raven. On the positive side, the predominant species were *Crinum firmifolium* Baker, *Ageratum conyzoides* L., *Mesosphaerum pectinatum* (L.) Kuntze, *Sacciolepis indica* (L.) Chase, *Alternanthera sessilis* (L.) R.Br. ex. DC., *Panicum brevifolium* L., *Leersia perrieri* ( *A. Camus* ) Laurent, *Scoparia dulcis* L., *Murdannia simplex* (Vahl) Brenan, and *Neptunia oleracea* Lour. The second axis (dbRDA2) which explained 23.3% of variance, captured a mix of relevés across the three sites. The species most represented in this axis were *Rhynchospora candida* (Ness) Boeckeler, *Oryza longistaminata* A. Chev. & Roehr., and *Cyanotis speciosa* (L.f.) Hassk.

**FIGURE 5:**
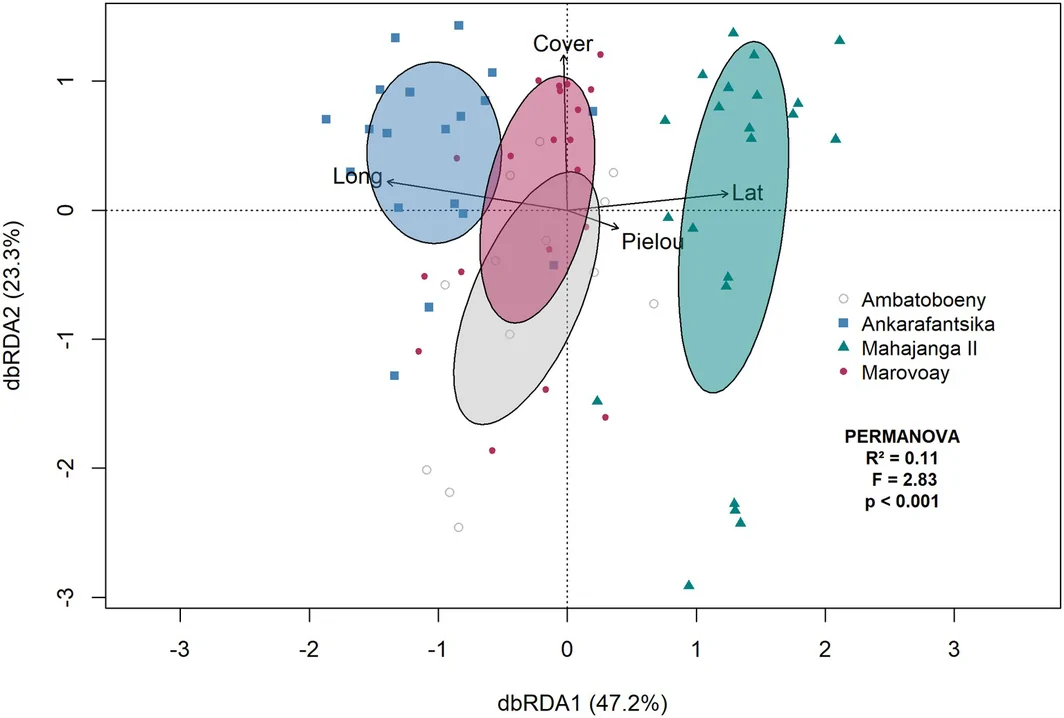
Distance‐based Redundancy Analysis (db‐RDA) with floristic variables (Cover: Total cover in 1 m^2^ plot); Pielou index, and geographical variables (Long: Longitude, and Lat: Latitude). A high negative correlation appears between longitude and latitude and Pielou index and longitude. Latitude and Longitude are also correlated with the first axis in positive and negative side, respectively, and the cover in the positive side in the axis 2. The Permutational Multivariate Analysis of Variance (PERMANOVA) shows that the floristic composition in each site is significantly different.

The PERMANOVA analysis exhibited significant differences among sites (*F* = 2.8; *p* = 0.001) and represented 10.8% of variation (*R* ^2^ = 10.8%). The pairwise PERMANOVA analysis revealed significant differences among the sites (*p* < 0.01, Figure 5). Notably, the samples from Mahajanga II were located on the positive side of axis 1, while the site from Ankarafantsika were located in the negative one. Then those from Marovoay, and Ambatoboeny were dispersed along axis 2.

### 3.6 Climatic Variation on the Study Sites

**FIGURE 6:**
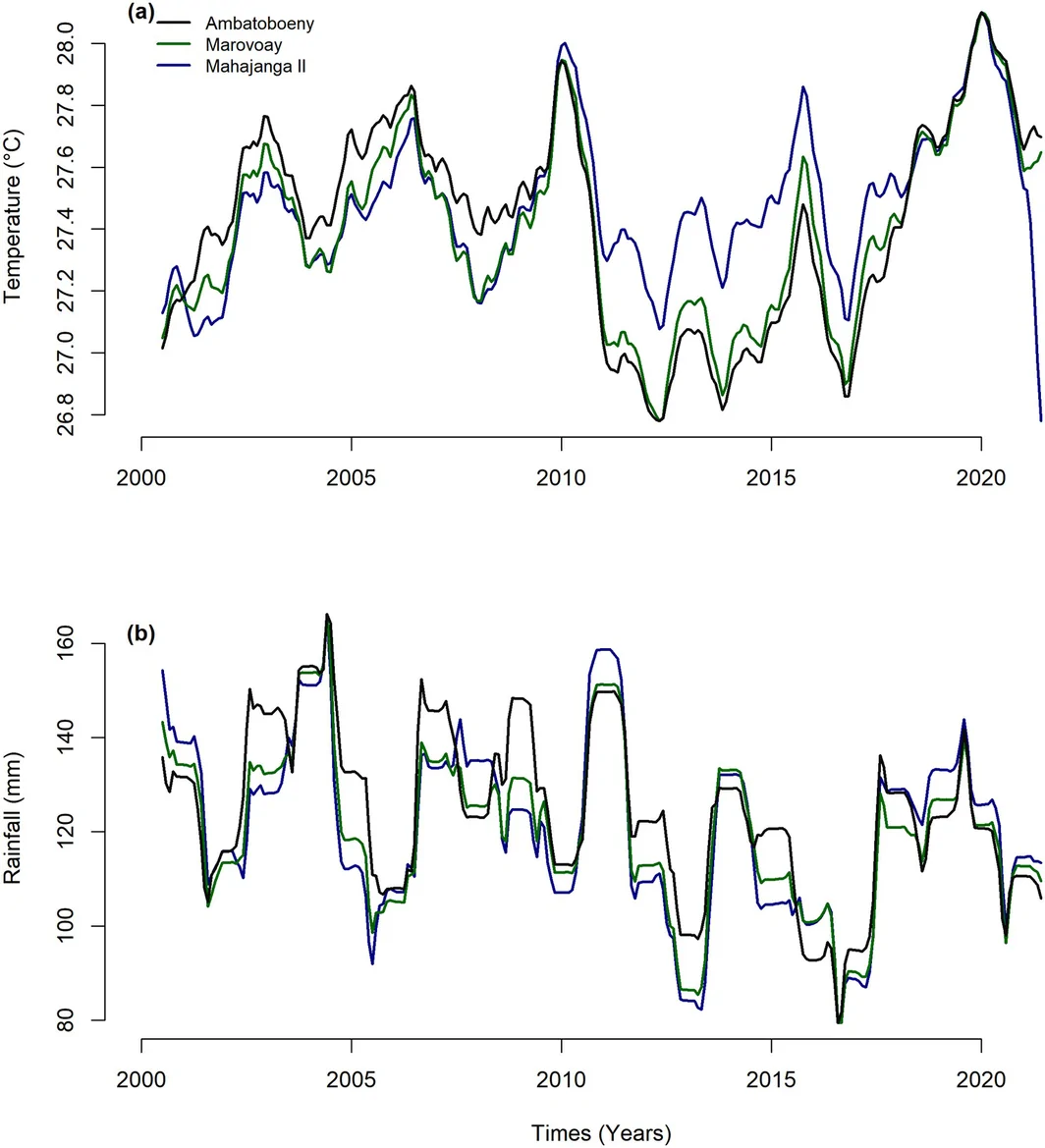
Trend of the climate variation in the three district as Ambatoboeny (black), Marovoay (Green), and Mahajanga II (Blue). (a) Temperature and (b) Rainfall from 2000 to 2021. The temperature in the study sites tend to increasing however, the rainfall tend to decrease over the two decades.

The temperature and rainfall data for the study sites: Ambatoboeny, Marovoay, and Mahajanga II illustrate climate variations over a period of 21 years, from 2000 to 2021 (Figure 6). The temperature trend peaked at approximately 28°C in 2010 across all three sites, increasing to 28.7°C by 2020. Between 2010 and 2015, Ambatoboeny and Marovoay experienced a relatively cooler period, while Mahajanga II saw warmer conditions. Precipitation levels reached around 171 mm but gradually decreased to about 113 mm in all three locations. In 2022, the temperature in the Mahajanga II district dropped to 26.8°C, with annual rainfall decreasing to less than 120 mm.

## 4 Discussion

### 4.1 Species Richness

The number of species varies from one site to another due to factors such as climate, soil types, topography, altitude, habitat types, and land use (Dar et al. 2014; Yang et al. 2022; Bomfim et al. 2023). Previous studies have also indicated that physical–chemical conditions of water significantly impact species dispersion, along with anthropogenic activities (Ranarijaona 2007; Phillipson et al. 2018). In total, 98 species were recorded across the four study sites, with 26.53% (26 species) comprising terrestrial plants that inhabit the banks of wetlands, and 73.47% (72 species) classified as freshwater plants, including helophytes and hydrophytes. In comparison, 77 species were documented in the peri‐urban areas of Mahajanga (Ramiandrisoa et al. 2017). However, in the eastern part of Madagascar, particularly in Lake Alaotra, Ranarijaona (2007) recorded 35 species, while Rakotoarivelo et al. (2019) identified 84 species, indicating a higher number of species compared to our study sites. Furthermore, in the southeastern region of Madagascar, 60 species were recorded in the district of Vohipeno (Ramiandrisoa 2018, not published). In South Africa, Du Toit et al. (2021) had recorded 102 species across the rural–urban gradient including the native and invasive species. However, in the Ethiopian wetlands, Fentaw et al. (2024) had only recorded 41 species of macrophytes in the Infranz and Wonjeta wetlands.

### 4.2 Diversity Indices

The rarefaction curve indicates that all lines have not yet reached a plateau, suggesting that the sample sizes are insufficient for each study site. In the case of Ambatoboeny and Mahajanga II, the wetlands are dispersed and cover relatively small areas. Conversely, at Ankarafantsika Park, samples were taken from Ravelobe Lake and the disturbed marshland. The most significant disturbed areas were found in the Marovoay wetlands, which have been converted into rice fields. The curve shows that the Ambatoboeny wetlands exhibit the highest values of Shannon diversity, indicating that some ponds experience low levels of disturbance. In contrast, the Mahajanga II wetlands face higher disturbance levels, particularly due to an extended period of drought. Although the Marovoay plain may initially have higher diversity, its conversion to rice fields has greatly disturbed the area, leading to a decrease in aquatic plant species richness. This type of conversion has been predominantly observed in the wetlands of northwestern Madagascar, as reported by Maharombaka et al. (2017).

### 4.3 Species Distribution

Some species have the ability to survive despite the various pressures on them and their habitat. Those that grow along the banks of the wetlands are easily accessible to herbivores. In contrast, species that are found in deeper areas may be uprooted by humans when wetlands are converted for agricultural use, as seen in the case of Marovoay, which has been transformed into a rice‐growing area. These disturbances lead to the limited distribution of endemic species. The rising temperatures and decreasing precipitation may lead to a decline in both the abundance and endemicity of macrophyte plant species. These species, hydrophytes, are especially vulnerable when wetlands become drier (Lind et al. 2022). The species *Panicum luridum* is not found in wetland habitat but is recorded on banks. It occurs at low to high altitudes (Vorontsova et al. 2018; Rabarivola et al. 2019; Ramiandrisoa et al. 2024). *Melochia betsiliensis*, which occurs up to 500 m.a.s.l., is found near wetlands; *Crinum firmifolium* a geophytic species present across Madagascar, can be found in forests and wetlands (Catalogue of Plants of Madagacar 2025; Catalogue of Plants of Madagascar 2025. http://legacy.tropicos.org/Name/30400126?projectid=17; http://legacy.tropicos.org/Name/1200358?projectid=17, respectively).

### 4.4 Biological Types

The biological classification provides information about the life forms of various species (Sirvent 2020). Helophytes, such as those in the Cyperaceae family, make up the majority of the recorded species. In deeper water areas, hydrophytes are predominant, particularly the nymphaeids (*Nymphaea* spp.). In contrast, terrestrial species like *Senna tora* and *Heliotropium indicum* can be found colonizing the banks and are also prevalent in pastures. In addition, some species are also found (i.e., common species from Madagascar) in the African continent belonging to the hydrophyte traits (e.g., floating speices) such as *Nymphaea lotus* L., *Pistia stratiotes* L. (Araceae), *Chara* sp. (Characeae), and emergent species like *Marsilea minuta* L. (Marsileaceae), *Cyperus rotundus* L., *Cyperus esculentus* L., *Pycreus macrostachyos* (Cyperaceae), *Ischaemum rugosum* Salisb., *Echinochloa colona* (L.) Link (Poaceae) (Fentaw et al. 2024; Behn et al. 2022).

### 4.5 Influence of the Floristic Distribution

The dbRDA analysis exhibited the geographical parameters (Longitude and Latitude) as a determinant of the species distribution. The Longitude corresponds to the locations of the Marovoay, Ambatoboeny and Ankarafantsika sample sites. The two first sites are interconnected by the Betsiboka river (Ambatoboeny and passed to Marovoay and dumped in the Bombetoka baie), and Ankarafantsika supply Marvoay plain from the Amboromalady artificial Lake. Most of the sites sampled in these areas were slightly disturbed except in some samples in the rice paddy. In the Ambatoboeny, samples were collected in the areas which had less disturbance in the ponds and for the areas in the rice paddy. In the case of Ankarafantsika, people lived around and had their own croplands in the south of Raveloba lake that enriched the species composition. In addition, people in the peripheral areas converted the wetlands (e.g., marshes) into croplands (e.g., rice paddy fields). During our investigation, many wetlands were not cultivated but remained abandoned and it allowed us to obtain an exceptional richness (45 species). The species recorded were typically found in the marshes such as *Pycreus mundtii* Nees, *Fuirena umbellata* Rottb. It's noted that the wetlands areas around the Ankarafantsika park are nearest to the rafia groves ( *Raphia farinifera* ) plantation that indicated a permanent source of freshwater. However, Raphia palms thrive in the wetlands (Kamta et al. 2021). In contrast, the species composition in the Mahajanga II site was influenced by the latitude. Despite the similarity of the usage of the wetlands (i.e., croplands), Mahajanga II had a temporary and semi‐temporary wetlands types. Like in the quasi‐totality of wetlands around the world (e.g., in Africa), this ecosystem is exploited by human and converted in agricultural fields (Khan et al. 2022) which confirms the vulnerability of the ecology of the aquatic plant species and their easiest threatened (Gardner et al. 2015).

The species recorded in these areas were typically found in the banks of the ponds and lakes like *Ageratum conyzoides* L., *Alternanthera sessilis* (L.) R.Br. Ex DC., *Mesosphaerum pectinatum* (L.) Kuntze. Some species were widely distributed such as *Rhynchospora candida* (Nees) Boeckeler. Another factor can participate in the differentiation of species composition (e.g., the nearest of the site from the sea, and the type of the substrates that we didn't consider as a factor). In fact, the soil characteristics (i.e., physical chemical) influence plant compositions (Infante Mata et al. 2011; Chmolowska et al. 2016). However, the main types of soil found in African wetlands, as well as in wetlands in other regions, are organic and mineral soils adapted to flooding and water saturation conditions. In Africa, there are peat soils (peatlands), which are rich in partially decomposed organic matter, as well as swamp and marsh soils composed of saturated minerals. These soils are often rich in iron and aluminium, such as the tropical plenthic soils found in regions like West Africa. Outside Africa, wetlands soils range from peatlands to various hydromorphic soils, such as Gleysoils, which are typical of permanently or seasonally saturated areas. Other wetland soils are characterized by permanent or temporary water saturation, acidity, and varying nutrient levels (Food and Agriculture Organization of the United Nations and Sub‐Regional Office for East and Southern Africa 1998; Schuijt and Fischer 2022; World reference base for soil resources 2006).

The distance‐based redundancy analysis revealed significant differences from Mahajanga II and the rest of the sites (Marovoay, Ambatoboeny, and Ankarafantsika). These differences in the species composition were due to the long distance between these sites. In contrast, the three other sites are geographically narrow in the plain forming Marovoay and Ambatoboeny district. These last sites are known for the greatest agricultural production in the Boeny Region, especially rice paddy for Marovoay and other cultivation for Ambatoboeny (CREAM 2013). The Ankarafantsika park is located in the overlapping districts.

### 4.6 Climate Variation on Sites

Climate hazards play an important role in biodiversity, the quantity of rainfall, and the variation of temperature in different regions around the world. The arid and semi‐arid climate areas were severely affected by the drought of wetlands (Lesk et al. 2016) that had a lot of consequences for the corresponding areas (e.g., crop production, wetlands biodiversity). According to Cornet (1970), the bioclimatic subdivision is characterized by the wind Alize from the East of Madagascar. The wind mass brings a high humidity to the high relief in the East and becomes drier as it descends the slope in the west catchment. Additionally, the anthropogenic activities (e.g., bush, savannas, and forest fires) contribute to rise the temperature. The increasing temperature cause a higher evapotraspiration to the vegetation, continental freshwater reserves. During our observations in the fields for a decade, the wetlands in the north west of the island show a drier period. Figure 6 demonstrates the decrease in the quantity of rainfall over the year. Then, globally, the northwestern part of Madagascar as well as the west coast, experienced a severely long period of drought. It may be linked to forest degradation and coverage loss, and repeated savanna burning (Hasina Andriamanantena et al. 2021; Frappier‐Brinton and Lehman 2022; Phelps et al. 2022) each year. In contrast, a natural disaster which brought a high quantity of rain reached the small period event in 2011 (Contribution au Processus National Global shield against Climate Risks 2025). According to Article 2 of the Paris Agreement (United Nations 2015) on climate change, keeping temperature increases below 2°C and no higher than 1.5°C is a cause for concern. However, temperatures in our study sites are close to this value despite the fact that Madagascar is not an industrialized country. At Marovoay, the minimum temperature is 26.71°C and the maximum of 27.78°C (27.27°C annual average), with an increase of +1.07°C, over the 20 years. Ramizason (2025, not published) observed a decrease in rainfall levels in the Marovoay district between 1981 and 2023, particularly after 2005. Temperature rose by +0.60°C between 2001 and 2010, and between 2017 and 2020. Then, in Mahajanga II, the minimum temperature was 26.73°C and the maximum of 27.88°C (27.43°C annual average), indicating an increase of +1.14°C. Finally, in Ambatoboeny, the minimum temperature was 26.22°C and the maximum of 27.57°C (26.97°C annual average) indicating an increase of +1.35°C.

## 5 Conclusion

Wetlands in the northwestern part of Madagascar are rich but highly vulnerable to climate hazards and human activities. Due to these disturbances, species are vulnerable and demand a particular attention for conservation. The distance between the study sites and the coverage of the species are the main factors that influenced the distribution of the floristic composition and their position around the wetlands (e.g., banks and inside the water).

## Funding

This work was supported by Re:Wild (SSC‐UICN) (Grant SMA‐G00‐00165).

## Conflicts of Interest

The authors declare no conflicts of interest.

## Supporting information


**Supporting Information: S1** List of species occuring in the whole study sites with their distributions.

**Supporting Information: S2** Boxplot showing the number of species in each zonation of the study site. The black dots are the number of relevés observed during the fieldwork, the bold line in the center represent the median of the species count. The first line below show the minimum number, the second and forth lines for the first quartile (Q1) and third quartile (Q3), respectively. Each panel represent the study site.

**Supporting Information: S3** Biological types of recorded species from the whole study sites.

## Data Availability

The data that supports the findings of this study are available in the Supporting Information of this article.
